# The concordance and discordance of diabetic kidney disease and retinopathy in patients with type 2 diabetes mellitus: A cross-sectional study of 26,809 patients from 5 primary hospitals in China

**DOI:** 10.3389/fendo.2023.1133290

**Published:** 2023-03-09

**Authors:** Zhaoxiang Liu, Xianglan Li, Yanlei Wang, Yanxia Song, Qiang Liu, Junxia Gong, Wenshuang Fan, Chunmei Lv, Chenxiang Cao, Wenhui Zhao, Jianzhong Xiao

**Affiliations:** ^1^ Beijing Tsinghua Changgung Hospital, School of Clinical Medicine, Tsinghua University, Beijing, China; ^2^ Department of Endocrinology, Beijing Ruijing Diabetes Hospital, Beijing, China; ^3^ Department of Endocrinology, Lanzhou Ruijing Diabetes Hospital, Lanzhou, China; ^4^ Department of Endocrinology, Taiyuan Diabetes Hospital, Taiyuan, China; ^5^ Department of Endocrinology, Heilongjiang Ruijing Diabetes Hospital, Harbin, China; ^6^ Department of Endocrinology, Chengdu Ryan Diabetes Hospital, Chengdu, China

**Keywords:** diabetic kidney disease, diabetic retinopathy, type 2 diabetes mellitus, metbolic syndrome, discordance

## Abstract

**Introduction:**

Diabetic kidney disease (DKD) and diabetic retinopathy (DR) share similar pathophysiological mechanisms. However, signs of DKD may be present at diagnosis of diabetes without retinopathy. Risk factors for the development of DKD and DR may not be identical.

**Methods:**

This study aimed to evaluate the concordance and discordance between DKD and DR by investigating the distribution of DKD and DR in patients with type 2 diabetes mellitus from 5 Chinese cities. A total of 26,809 patients were involved in this study. The clinical characteristics were compared among patients based on the presence of DKD and DR. Logistic regression models were used to analyze the independent risk factors of DKD and DR.

**Results:**

The prevalence of DKD and DR was 32.3% and 34.6%, respectively. Among eligible patients, 1,752 patients without DR had an increased urinary albumin-to-creatinine ratio (ACR) or reduced estimated glomerular filtration rate (eGFR), and 1,483 patients with DR had no DKD. The positive predictive value of DR for DKD was 47.4% and negative predictive value was 67.1%. Elder age, male gender, a longer duration of disease, higher values of waist circumference and HbA1c were associated with both DR and DKD. A lower educational level was associated with DR. Higher BP and TG would predict increased prevalence of DKD.

**Conclusions:**

DKD and DR shared many risk factors, but a significant discordance was present in patients with type 2 diabetes mellitus. DKD was more strongly associated with blood pressure and triglycerides than DR.

## 1 Introduction

Diabetic kidney disease (DKD) affects 20-40% of patients with diabetes (1, 2). The prevalence of DKD or chronic kidney disease (CKD) in Chinese patients with diabetes is increasing (3, 4) as type 2 diabetes mellitus becomes an epidemic disease. DKD is diagnosed based on the presence of albuminuria and/or the reduced estimated glomerular filtration rate (eGFR < 60 mL/min/1.73m^2^) in the absence of signs or symptoms of other kidney diseases. Previous studies suggested that DKD might not solely develop from microalbuminuria to macroalbuminuria to azotemia as Mogensen proposed (5, 6). The reduced eGFR without albuminuria has been frequently reported in patients with type 1 diabetes mellitus and type 2 diabetes mellitus (7).

Diabetic retinopathy (DR), another microvascular complication, is supposed to share similar pathophysiological mechanisms with DKD, and the two are frequently found simultaneously. El-Asrar et al. reported that type 1 diabetes mellitus patients with DR were 13.39 times more likely to develop DKD than those without DR (8). Results of a meta-analysis showed that patients with DR were nearly 4 times more likely to be complicated by DKD. Patients with DKD were twice more likely to be diagnosed as DR (9). DR was typically used as an indicator of DKD in the differential diagnosis (10). However, discordance of DKD and DR was also discussed. Signs of DKD may be present in the time of diagnosis or in type 2 diabetes mellitus patients without retinopathy (11). It was reported that risk factors for the development of DKD and DR might not be identical. Japanese scholars demonstrated that systolic blood pressure (SBP) variability was an independent predictor for the development and progression of DKD, rather than DR, in type 2 diabetes mellitus patients (12). Genetic data revealed that the DR-related single nucleotide polymorphisms did not have an individual or cumulative genetic effect on the risk of DKD, eGFR status or end-stage renal disease (ESRD) outcomes of type 2 diabetes mellitus patients in Taiwan (13). The most important evidence originates from a series of randomized controlled trials published in recent years (i.e., new classes of antidiabetic drugs have different preventive effects on DKD and DR) (14). A meta-analysis showed that hypoglycemic medicine glucagon-like peptide 1 receptor agonists (GLP-1RA) reduced the risk of kidney disease progression by 18% (hazard ratio (HR), 0.82, 95% confidence interval (CI): 0.75-0.89, *P*<0.001), while sodium-glucose cotransporter 2 (SGLT2) inhibitors reduced the mentioned risk by 38% (HR, 0.62, 95%CI, 0.58-0.67, *P*<0.001) (15). The preventive effects of GLP-1RA and SGLT2 inhibitors on DR in humans have not yet been reported (14). Taken together, the concordance and discordance of DKD and DR in patients with type 2 diabetes mellitus exist and need to be further elaborated.

The present study aimed to investigate the concordance and discordance between DKD and DR, as well as the relevant risk factors.

## 2 Methods

### 2.1 Study subjects

**Figure 1:**
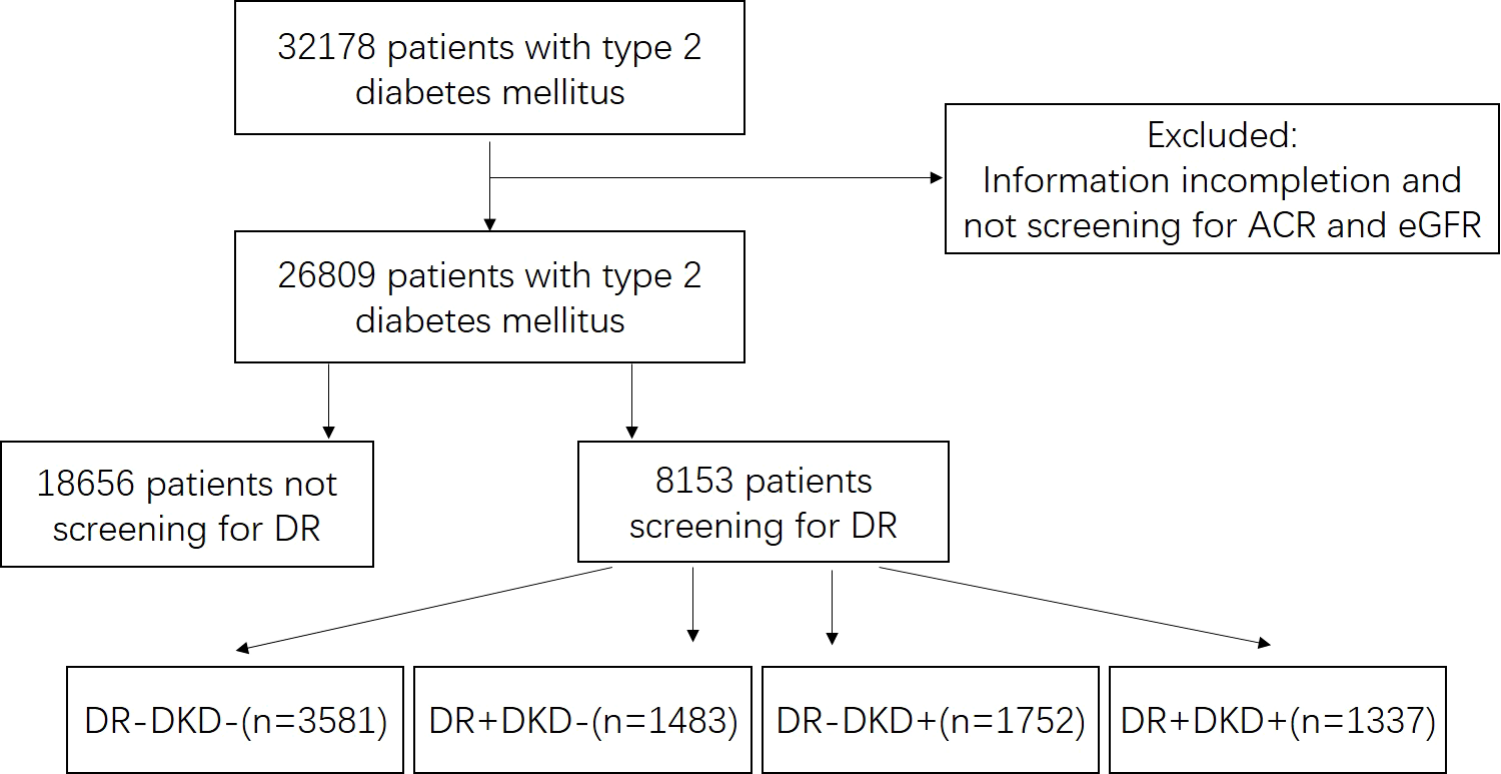
Flowchart of screening patients. ACR, urinary albumin-to-creatinine ratio. EGFR, estimated glomerular filtration rate. DR, diabetic retinopathy. “+” means positive, and “-” means negative.

Patients with type 2 diabetes mellitus who were admitted to Ruijing diabetes hospital chains (China) were enrolled in this study. Five hospitals from Beijing, Lanzhou, Harbin, Chengdu, and Taiyuan were included. The data were collected continuously from March 2016 to December 2021. The inclusion criteria were as follows: diagnosis of type 2 diabetes mellitus was based on the diagnostic criteria presented by the World Health Organization (WHO) in 1999 (16), and patients who aged 18 - 80 years old. Those patients who had severe heart (New York Heart Association III/IV), liver (severe hepatic impairment or liver failure), lung (conditions that may predispose to hypoxemia), or renal diseases (primary nephrotic syndrome, glomerulonephritis, obstructive renovascular disease, nephrectomy, renal transplant, etc.), and those were pregnant, or had been diagnosed with type 1 diabetes mellitus, special type of diabetes or gestational diabetes were excluded. This study was approved by the Ethics Committee of Tsinghua Changgung Hospital (Beijing, China; Approval No. [2016] 004). The flowchart of screening patients was shown in **Figure 1**.

### 2.2 Data collection

Patients’ data were collected at the first visit in each hospital through face-to-face interviews, including demographic data, educational level, smoking status, individual medical history (hypertension, dyslipidemia, and cardiovascular disease), and family history of diabetes mellitus. Blood samples were collected after an overnight (10-14 h) fasting, and the laboratory tests were conducted in the local hospital, including liver function, renal function, fasting plasma glucose (FPG), glycosylated hemoglobin (HbA1c), and lipid profiles (low-density lipoprotein cholesterol (LDL-C), high-density lipoprotein cholesterol (HDL-C), triglyceride (TG)). HbA1c was measured by high-performance liquid chromatography using the ADAMS A1c, HA-8180T analyzer (Array, Tokyo, Japan) or MQ-2000 PT Balk analyzer (Huazhong, Shanghai, China), which was the second-level reference for glycosylated hemoglobin of International Clinical Chemistry Committee. Blood lipids, liver functions and kidney functions were assessed by automated analysis (AU5800; Beckman Coulter Inc., Brea, CA, USA). Urinary albumin was determined using a DADE BEHRING BN II analyzer (Siemens, Munich, Germany) by nephelometry (N antiserum to Human Albumin Assay, Dade Behring). Urinary creatinine concentration was measured *via* a Hitachi 7600 analyzer (Hitachi, Tokyo, Japan) using the sarcosine oxidase-PAP method. The urinary albumin-to-creatinine ratio (ACR) was computed and was reported in milligrams per gram (mg/g). Retinopathy status was assessed by fundus photography (TRC-NW100 camera; Nikon, Tokyo, Japan), and all images were graded by an experienced ophthalmologist. Diagnostic criteria of DR were based on the worse eye according to international clinical diabetic retinopathy and diabetic macular edema disease severity scales published in 2002 (17). All the laboratories participated in the quality control program as requested by the authority. All data were automatically downloaded from hospital information system.

DKD was defined as elevated urinary ACR (≥30 mg/g), or reduced eGFR (<60 mL/min/1.73 m^2^), or both, for longer than 3 months, excluding clinically significant renal diseases through medical history and laboratory results, in accordance with the current guidelines of Kidney Disease: Improving Global Outcomes (KDIGO) (18, 19). The eGFR was calculated using the Modification of Diet in Renal Disease (MDRD) study formula (20) as follows: 186 × [serum creatinine (mg/dL)] - 1.154×(age) - 0.203 × (0.742 if female). The diagnosis of albuminuria was divided into three stages according to ACR (ACR < 30 mg/g was defined as non-albuminuria, 30 mg/g ≤ ACR < 300 mg/g as microalbuminuria, and ACR ≥ 300 mg/g as macroalbuminuria).

Diagnosis of metabolic syndrome was made by presence of any three or more of the following (21): 1. Abdominal obesity (central obesity): waist circumstance ≥90 cm in men or ≥85cm in women. 2. Hyperglycaemia: FPG ≥ 6.1 mmol/L or OGTT 2hPG ≥ 7.8 mmol/L and/or confirmed diabetes that is under treatment. 3. Hypertension: blood pressure ≥130/85 mmHg and/or diagnosed and on antihypertensive therapy. 4. Fasting TG ≥ 1.70 mmol/L. 5. Fasting HDL‐C < 1.04 mmol/L.

### 2.3 Statistical analysis

SPSS 23.0 software (IBM, Armonk, NY, USA) was used for data analysis. Normally distributed data were expressed as the mean ± standard deviation (SD), and abnormally distributed data were expressed as median (interquartile range). The χ^2^ test was used to compare the clinical categorical variables among different groups. Logistic regression models were established to analyze the independent risk factors of DKD and DR. Risk factors included age (every 10 years), gender (female as 0, male as 1), duration of disease (every 5 years), educational level (junior school or below as 1, high school or above as 2), body mass index (BMI, < 24 kg/m^2^ as 1, ≥24 kg/m^2^ as 2), waist circumference (every 10 cm), smoking history (never as 0, with smoking history as 1), HbA1c (< 7% as 1, 7% ~ 9% as 2, ≥ 9% as 3), systolic blood pressure (SBP, <140 mmHg as 1, 140mmHg ~ 160 mmHg as 2, ≥160 mmHg as 3), LDL-C (< 2.6 mmol/L as 1, 2.6 mmol/L ~ 3.3 mmol/L as 2, ≥ 3.3 mol/L as 3), TG (< 1.7 mmol/L as 1, 1.7 mmol/L ~ 5.0 mmol/L as 2, ≥5.0mol/L as 3), and DR (absent as 0, non-proliferative retinopathy (NPDR) as 1, and proliferative retinopathy (PDR) as 2). *P <*0.05 indicated statistical significance.

## 3 Results

### 3.1 Clinical characteristics of patients with type 2 diabetes mellitus

A total of 26,809 patients with type 2 diabetes mellitus were involved in this study. There were 14,813 (55.3%) male patients and 11,996 (44.7%) female patients. The average age, duration of disease, BMI, and HbA1c were 59.2 ± 10.7 years old, 8.6 ± 6.9 years, 25.2 ± 3.4 kg/m^2^, and 8.6 ± 2.1% (70 mmol/mol), respectively.

Data of ACR and eGFR were available for all patients. There were 18,875 (70.4%) patients with eGFR ≥ 90mL/min/1.73m^2^, 6,685 (24.9%) patients with eGFR equal to 60-90 mL/min/1.73m^2^, 1,053 (3.9%) patients with eGFR equal to 30-60 mL/min/1.73m^2^, and 196 (0.7%) patients with eGFR < 30 mL/min/1.73m^2^. The majority of patients had normal albuminuria level (69.1%), and 23.5% and 7.4% of them had microalbuminuria or macroalbuminuria, respectively. According to the latest diagnostic criteria for DKD, there were 8,660 (32.3%) patients with eGFR < 60 mL/min/1.73m^2^ and/or ACR ≥ 30 mg/g, including 384 (1.4%) patients without albuminuria (eGFR < 60 mL/min/1.73m^2^ and ACR < 30 mg/g), 7,411 (27.6%) patients with eGFR ≥ 60 mL/min/1.73m^2^ and ACR ≥ 30 mg/g, and 865 (3.2%) patients with eGFR < 60 mL/min/1.73m^2^ and ACR ≥ 30 mg/g.

Among 8,153 patients who were screened for retinopathy status, there were 2,820 (34.6%) patients who were diagnosed with DR, including 2,592 patients with NPDR and 228 patients with PDR. Comparison between DKD negative (n = 5064) with DKD positive (n = 3089) and DR negative (n = 5333) with DR positive (n = 2820) were made in those people (**Table 1**). Patients with DKD were elder, had longer duration of disease, higher values of BMI, waist circumference, HbA1c, BP, LDL-C, TG, and higher proportion of metabolic syndrome than those with DKD-negative. Similar significant clinical indicators were observed in patients with DR compared to those without, except for BMI and TG.

**Table 1:** Characteristics of different groups of patients with DKD negative, DKD positive, DR negative and DR positive.

	All (n=8153)	DKD negative (n=5064)	DKD positive (n=3089)	*P*	DR negative (n=5333)	DR positive (n=2820)	*P*
**Age (years)**	59.45 ± 10.51	58.94 ± 10.30	60.28 ± 10.81	<0.001	58.95 ± 10.94	60.39 ± 9.60	<0.001
**Duration of disease (years)**	8.80 ± 6.89	8.12 ± 6.53	9.93 ± 7.30	<0.001	7.97 ± 6.64	10.39 ± 7.07	<0.001
**BMI (kg/m^2^)**	25.24 ± 3.38	25.09 ± 3.30	25.48 ± 3.50	<0.001	25.18 ± 3.38	25.34 ± 3.39	0.055
**Waist circumference (cm)**	90.14 ± 9.45	89.62 ± 9.12	90.99 ± 9.82	<0.001	89.86 ± 9.40	90.69 ± 9.52	<0.001
**Fasting blood glucose**	10.86 ± 4.11	10.38 ± 3.92	11.62 ± 4.29	<0.001	10.69 ± 4.07	11.21 ± 4.18	0.001
**HbA1c (%)**	8.82 ± 2.14	8.63 ± 2.09	9.15 ± 2.18	<0.001	8.74 ± 2.17	8.98 ± 2.07	<0.001
**SBP (mmHg)**	134.45 ± 18.42	131.78 ± 16.99	138.82 ± 19.79	<0.001	133.71 ± 18.29	135.85 ± 18.59	<0.001
**DBP (mmHg)**	80.51 ± 11.11	79.51 ± 10.63	82.16 ± 11.66	<0.001	80.21 ± 11.02	81.10 ± 11.25	0.001
**LDL-c (mmol/L)**	2.73 (2.17,3.33)	2.72 (2.17,3.30)	2.76 (2.17,3.38)	<0.001	2.72 (2.16,3.30)	2.77 (2.20,3.37)	0.003
**TG (mmol/L)**	1.70 (1.20,2.49)	1.62 (1.17,2.34)	1.81 (1.30,2.70)	<0.001	1.70 (1.20,2.50)	1.70 (1.21,2.47)	0.249
**HDL-c (mmol/L)**	1.34 (1.15,1.56)	1.34 (1.16,1.56)	1.34 (1.14,1.57)	0.683	1.34 (1.16,1.57)	1.33 (1.15,1.56)	0.342
**Smoking history**	10.7%	10.2%	11.5%	0.148	10.2%	11.6%	0.086
**Utilization rate of ACEI/ARB**	25.7%	21.7%	32.3%	<0.001	22.0%	32.7%	<0.001
**Rate of HbA1c <7%**	21.1%	24.0%	16.5%	<0.001	23.2%	17.2%	<0.001
**Rate of BP < 130/80 mmHg**	22.2%	25.4%	17.1%	<0.001	23.1%	20.6%	0.008
**Rate of LDL-c < 2.6 mmol/l**	43.6%	44.5%	42%	0.029	44.2%	42.4%	0.130
**eGFR (mL/min/1.73m^2^)**	95.53 ± 19.71	98.80 ± 16.93	90.18 ± 22.57	<0.001	96.60 ± 20.17	93.52 ± 18.65	<0.001
**ACR (mg/g)**	16.90 (5.90,61.92)	8.00 (3.65,15.00)	106.71 (48.60,305.40)	<0.001	14.10 (5.22,14.10)	24.48 (7.93,133.18)	<0.001
**Proportion of metabolic syndrome**	71.9%	68.4%	77.7%	<0.001	70.7%	74.1%	0.001

DKD negative: ACR < 30mg/g and eGFR > 60 mL/min/1.73m^2^; DKD positive: ACR < 30mg/g or eGFR > 60 mL/min/1.73m^2^; DR negative: normal fundus; DR positive: non-proliferative retinopathy (NPDR) and proliferative retinopathy (PDR).

There were 3,581 patients with DR-negative and DKD-negative, 1,483 patients with DR-positive and DKD-negative, 1,752 patients with DR-negative and DKD-positive, and 1,307 patients with DR-positive and ACR-positive. Patients’ clinical characteristics in the four groups are shown in **Table 2**. For patients with DR-positive and DKD-positive, they had the longest duration of disease, the highest HbA1c, BP, and TG level, the lowest eGFR, and the highest ACR. DR was more frequent in patients with DKD, while it was not an indicator of DKD. The positive predictive value (PPV) of DR for DKD was 47.4% and negative predictive value (NPV) was 67.1%.

**Table 2:** Characteristics of different groups of patients with DR-DKD-, DR-DKD+, DR+DKD- and DR+DKD+.

	DR-DKD- (n=3581)	DR+DKD- (n=1483)	DR-DKD+ (n=1752)	DR+DKD+ (n=1337)	*P*
**Age (years)**	58.43±10.63	60.17±9.35^*^	60.02±11.47^*^	60.63±9.86^*^	<0.001
**Duration of disease (years)**	7.53±6.36	9.53±6.74^*^	8.85±7.11^*#^	11.35±7.31^*#†^	<0.001
**BMI (kg/m^2^)**	25.05±3.31	25.17±3.27	25.45±3.49^*#^	25.52±3.51^*#^	<0.001
**Waist circumference (cm)**	89.40±9.20	90.17±9.10^*^	90.79±9.72^*^	91.25±9.94^*#^	<0.001
**Fasting blood glucose**	10.33±3.93	10.50±3.91	11.36±4.24^*#^	12.00±4.33^*#†^	<0.001
**HbA1c (%)**	8.60±2.14	8.69±1.97	9.04±2.21^*#^	9.30±2.14^*#†^	<0.001
**SBP (mmHg)**	131.51±17.00	132.45±16.88	138.21±19.89^*#^	139.61±19.65^*#†^	<0.001
**DBP (mmHg)**	79.30±10.60	80.01±10.69^*^	82.05±11.62^*#^	82.30±11.72^*#^	<0.001
**LDL-c (mmol/L)**	2.70(2.17,3.28)	2.75(2.19,3.33)	2.75(2.14,3.34) ^*^	2.80(2.21,3.41) ^*#^	<0.001
**TG (mmol/L)**	1.63(1.17,2.36)	1.60(1.17,2.30)	1.82(1.27,2.77) ^*#^	1.80(1.30,2.61) ^*#†^	<0.001
**HDL-c (mmol/L)**	1.34(1.16,1.57)	1.33(1.16,1.55)	1.34(1.15,1.57)	1.34(1.14,1.57)	0.803
**Smoking history**	10.1%	10.5%	10.4%	12.9%^*#†^	0.040
**Utilization rate of ACEI/ARB**	19.5%	27.0%^*^	27.2%^*^	39.0%^*#†^	<0.001
**Rate of HbA1c <7%**	25.5%	20.4%^*^	18.6%^*^	13.8%^*#†^	<0.001
**Rate of BP < 130/80 mmHg**	25.9%	24.1%	17.4%^*#^	16.7%^*#^	<0.001
**Rate of LDL-c < 2.6 mmol/l**	45.0%	43.2%	42.4%	41.5%^*^	0.094
**eGFR (mL/min/1.73m^2^)**	99.27±18.33	97.67±12.89^*^	91.14±22.54^*#^	88.92±22.57^*#†^	<0.001
**ACR (mg/g)**	7.70(3.50,15.00)	8.50(4.00,15.40)	85.40(44.86,249.15) ^*#^	146.70(57.90,374.13) ^*#†^	<0.001
**Proportion of metabolic syndrome**	67.7%	70.0%	76.9%^*#^	78.7%^*#^	<0.001

^*^ compared with patients of DR-DKD-, P < 0.05. ^#^ compared with patients of DR+DKD-, P < 0.05. ^†^ compared with patients of DR-DKD-, P < 0.05.

### 3.2 Concordance and discordance between DR and DKD

Logistic regression models were established to estimate risk factors for DKD and DR, respectively. Elder age, male gender, a longer duration of disease, higher values of waist circumference and a higher HbA1c level were associated with both DKD and DR. A lower educational level was associated with DR. Higher BP and TG would predict increased prevalence of DKD (**Table 3**).

**Table 3:** Comparison of predictors for DKD and DR in all patients (n = 7409).

	DKD (n = 2783 )			DR (n = 2525)		
	β	OR(95% confidence interval)	*P*	β	OR(95% confidence interval)	*P*
**Age (years)**	0.108	1.114(1.06,1.17)	<0.001	0.060	1.062(1.01,1.117)	0.018
**Gender (M vs F)**	0.107	1.113(1.002,1.236)	0.045	0.163	1.177(1.058,1.31)	0.003
**Duration (years)**	0.222	1.248(1.177,1.324)	<0.001	0.373	1.452(1.367,1.543)	<0.001
**Educational level**	-0.043	0.958(0.867,1.058)	0.399	-0.477	0.621(0.562,0.686)	<0.001
**BMI (kg/m^2^)**	0.021	1.021(0.911,1.145)	0.720	0.012	1.012(0.901,1.136)	0.845
**Waist circumference (cm)**	0.080	1.084(1.023,1.148)	0.006	0.061	1.063(1.003,1.127)	0.040
**Smoking history**	0.140	1.15(0.976,1.355)	0.096	0.102	1.108(0.938,1.308)	0.229
**HbA1c (%)**	0.325	1.384(1.297,1.476)	<0.001	0.211	1.235(1.157,1.318)	<0.001
**SBP (mmHg)**	0.474	1.606(1.483,1.739)	<0.001	0.071	1.073(0.989,1.165)	0.090
**LDL-c (mmol/L)**	0.045	1.046(0.985,1.111)	0.146	0.053	1.054(0.992,1.121)	0.091
**TG (mmol/L)**	0.316	1.371(1.26,1.491)	<0.001	-0.048	0.953(0.874,1.039)	0.277

Risk factors for diabetes mellitus complicated by an increased ACR versus a reduced eGFR and NPDR versus DR were shown in supplemental materials (**Supplementary Tables 1**, **2**). They shared most risk factors.

## 4 Discussion

It was found that 32.3% and 34.6% of Chinese patients with type 2 diabetes mellitus were complicated by DKD and DR, respectively. Hospital-based investigation and the longer duration of diabetes may be explanation for the difference with that reported previously (22).

As we know, both DR and DKD are microvascular complications of diabetes mellitus. Evidence-based medical research showed that lowering blood glucose and blood pressure reduced the incidence rates of DKD and DR (23). Because these two complication are tightly correlated, DR is often used in clinical practice to differentiate DKD from other CKDs (24). However, retinopathy was absent in 56.7% of patients with DKD in this study. In contrast, 52.6% of patients with retinopathy did not have DKD. The discordance was 39.7% (DR-negative and DKD-positive plus DR-positive and DKD-negative) in the present study. A similar finding was reported in an Italian study, the discordance between DR and DKD was 36.6% (25). Interestingly, data from a real-world study revealed that there was no significant difference in albumin excretion rate between the presence and absence of DR in the whole population (26).

The estimated PPV of DR for DKD was 47.4% in this study, and the NPV of DR for DKD was 67.1%. This PPV was lower than the reports from KDIGO, i.e., the PPV of retinopathy for typical diabetic glomerulopathy ranged from 67% to 100% in patients with macroalbuminuria, and the NPV had a broader range of 20-84%. For microalbuminuria, PPVs were lower at around 45%, while NPVs were close to 100% (27). The prevalence of DKD was about 60% in patients with type 2 diabetes mellitus with advanced DR (28). A meta-analysis demonstrated that the pooled sensitivity and specificity of DR to predict DKD were 0.65 and 0.75, respectively, while PDR had a low sensitivity (0.25) and high specificity (0.98) for predicting DKD, respectively (10). Taken together, these results suggested that DR was not sensitive enough to predict DKD but it was good indicator to confirm DKD.

Elder age, male gender, a longer duration of disease, a higher value of waist circumference and a higher HbA1c level were correlated with both DKD and DR. These findings were also confirmed in other previous studies (29, 30). Although DKD and DR share similar mechanisms, numerous studies suggested that DKD and DR may differ in some way. Firstly, a noticeable proportion of patients with type 2 diabetes mellitus had DKD or DR alone (25). Secondly, a new classification of diabetes had been proposed by a Swedish group according to GAD antibody, BMI, age at onset, HbA1c level, homeostatic model assessment-β (HOMA-β), and HOMA of insulin resistance (HOMA-IR). Among them, cluster 3 (characterized by severe insulin resistance) was related to a higher incidence of kidney disease and cardiovascular disease, while cluster 2 (characterized by severe insulin deficiency) was associated with a higher incidence of DR (31). More importantly, SGLT2 inhibitors and GLP1-RA, two new classes of hypoglycemic drug, exerted outstanding renal but not retinal protective effects (32). Therefore, it was reasonable to assume that DKD and DR would be associated with different risk factors and pathogeneses.

Metabolic syndrome was called insulin resistance syndrome. In the present study, the proportion of metabolic syndrome was much higher in patients with DKD than those in DKD negative group. The higher level of SBP and triglyceride were independently associated with DKD but not DR. These were consistent with the new classification according to cluster analysis (31), i.e., patients with severe insulin resistance were more likely to be complicated by kidney disease. A study demonstrated that the visceral adiposity index was found to be strongly associated with the prevalence of DKD, while it was not associated with the prevalence of DR in Chinese subjects (33). Taken together, these data suggested that improving insulin resistance as well as controlling metabolic syndrome in patients with type 2 diabetes mellitus may be much more important in the prevention of DKD, as compared to in the prevention of DR.

We found that patients with DR were associated with lower educational levels. It was reported that patients with higher educational level may be prone to internalize health information and hence change their life-style, which could explain for lower DR rate in those patients (34).

The present study had several limitations. Firstly, the cross-sectional nature of this study precluded exploration of any cause-effect relationship. Secondly, concomitantly treatment affected the measurement of HbA1c, SBP, triglycerides and other biologic parameters. Thirdly, DKD was diagnosed based only on the clinical characteristics without renal biopsy, so that DKD might be over diagnosed. False positive of increased ACR due to poor blood glucose control may also be a concern. Last but not least, the proportion of screening of DR in patients with proteinuria was 36.2%, while it was 27.8% in patients without proteinuria. Patients with albuminuria were more likely to screen their retinopathy status, which might lead to selection bias. More detailed and comprehensive screening of DR are needed for Chinese patients with diabetes. The strength of this study was its large sample size. All participants were from 5 cities in China, and the large sample size might promote the generalization of the findings. The concordance and discordance between DR and DKD were discussed, and the corresponding strategies were put forward for the prevention of DKD.

## 5 Conclusion

The discordance was significant between retinopathy and DKD in type 2 diabetes. DKD was associated with a higher level of components of the metabolic syndrome, DR was more in patients with lower educational level. Further studies are required to discriminate their differences in the development and prevention of DR and DKD.

## Guarantor statement

JX is the guarantor of this work and had full access to all the data in the study and takes responsibility for the integrity of the data and the accuracy of the data analysis.

## Data availability statement

The original contributions presented in the study are included in the article/**Supplementary Material**. Further inquiries can be directed to the corresponding author.

## Ethics statement

The studies involving human participants were reviewed and approved by Ethics Committee of Tsinghua Changgung Hospital. The patients/participants provided their written informed consent to participate in this study.

## Author contributions

ZL conceptualized the study, interpreted the analyses, wrote the initial manuscript, and reviewed and revised the manuscript. XL collected data, contributed intellectually to the research topics, and critically reviewed the scientific content of the manuscript. YW analyzed the data, designed and supervised the statistical analysis, and reviewed and revised the manuscript. YS, QL, JG, WF, and CL collected data, reviewed and revised the manuscript. CC and WZ conceptualized the study, supervised the statistical analysis, and reviewed and revised the manuscript. JX conceptualized the study, coordinated and supervised data collection, acquired funding for the study, and critically reviewed the manuscript for important intellectual content. JX is the guarantor of this research and, as such, had full access to all the data in the study and takes responsibility for the integrity of the data and the accuracy of the data analysis. All authors contributed to the article and approved the submitted version.
