# Supplementary material for: The concordance and discordance of diabetic kidney disease and retinopathy in patients with type 2 diabetes mellitus: A cross-sectional study of 26,809 patients from 5 primary hospitals in China
Source: Front Endocrinol (Lausanne). 2023 Mar 9;14:1133290. doi: 10.3389/fendo.2023.1133290 (PMC10034101; doi:10.3389/fendo.2023.1133290)
Supplement: Supplementary file 1 [file DataSheet_1.docx]

**Supplemental Material**

Table1. Predictors for eGFR＜60 mL/min/1.73m^2^ or ACR ≥ 30 mg/g in patients with diabetes (n = 7409).

|  | eGFR＜60 mL/min/1.73m^2^ (n = 345) | | | ACR ≥ 30 mg/g (n = 2703) | | |
| --- | --- | --- | --- | --- | --- | --- |
|  | **β** | **OR(95% confidence interval)** | ***P*** | **β** | **OR(95% confidence interval)** | ***P*** |
| Age (years) | 0.649 | 1.914(1.669,2.194) | ＜0.001 | 0.065 | 1.067(1.015,1.121) | 0.010 |
| Gender (M vs F) | -0.050 | 0.951(0.75,1.207) | 0.681 | 0.127 | 1.136(1.021,1.263) | 0.019 |
| Duration of disease (years) | 0.365 | 1.441(1.248,1.664) | ＜0.001 | 0.184 | 1.201(1.131,1.276) | ＜0.001 |
| Educational level | -0.124 | 0.884(0.705,1.107) | 0.283 | 0.006 | 1.006(0.909,1.114) | 0.901 |
| BMI (kg/m^2^) | -0.161 | 0.851(0.659,1.099) | 0.217 | 0.012 | 1.012(0.901,1.137) | 0.835 |
| Waist circumference (cm) | -0.029 | 0.972(0.851,1.11) | 0.673 | 0.075 | 1.078(1.017,1.142) | 0.012 |
| Smoking history | 0.043 | 1.044(0.692,1.576) | 0.837 | 0.094 | 1.099(0.93,1.297) | 0.267 |
| HbA1c (%) | -0.257 | 0.773(0.669,0.894) | 0.001 | 0.322 | 1.38(1.292,1.474) | ＜0.001 |
| SBP (mmHg) | 0.368 | 1.445(1.233,1.692) | ＜0.001 | 0.478 | 1.613(1.488,1.748) | ＜0.001 |
| LDL-c (mmol/L) | 0.189 | 1.208(1.055,1.383) | 0.006 | 0.032 | 1.033(0.972,1.098) | 0.299 |
| TG (mmol/l) | 0.666 | 1.947(1.611,2.354) | ＜0.001 | 0.307 | 1.36(1.249,1.481) | ＜0.001 |
| DR (present vs. absent) | 0.465 | 1.593(1.269,1.998) | ＜0.001 | 0.482 | 1.619(1.477,1.775) | ＜0.001 |

Table2 Comparison of predictors for non-proliferative retinopathy (NPDR, total n = 7213) and DR (NPDR plus proliferative retinopathy (PDR), total n = 7409)

|  | NPDR (n = 2329) | | | DR (NPDR + PDR, n = 2525) | | |
| --- | --- | --- | --- | --- | --- | --- |
|  | **β** | **OR(95% confidence interval)** | ***P*** | **β** | **OR(95% confidence interval)** | ***P*** |
| Age (years) | 0.086 | 1.09(1.031,1.152) | 0.002 | 0.059 | 1.061(1.006,1.119) | 0.029 |
| Gender (M vs F) | 0.165 | 1.18(1.057,1.317) | 0.003 | 0.146 | 1.157(1.039,1.288) | 0.008 |
| Duration (years) | 0.346 | 1.413(1.327,1.504) | ＜0.001 | 0.342 | 1.408(1.325,1.497) | ＜0.001 |
| Educational level | -0.461 | 0.631(0.569,0.7) | ＜0.001 | -0.473 | 0.623(0.563,0.69) | ＜0.001 |
| BMI (kg/m^2^) | -0.005 | 0.995(0.882,1.122) | 0.934 | 0.019 | 1.02(0.907,1.147) | 0.746 |
| Waist circumference (cm) | 0.058 | 1.06(0.998,1.126) | 0.060 | 0.052 | 1.053(0.993,1.117) | 0.084 |
| Smoking history | 0.105 | 1.111(0.935,1.318) | 0.231 | 0.084 | 1.087(0.919,1.287) | 0.331 |
| HbA1c (%) | 0.167 | 1.181(1.104,1.265) | ＜0.001 | 0.171 | 1.187(1.111,1.269) | ＜0.001 |
| SBP (mmHg) | -0.008 | 0.992(0.91,1.081) | 0.856 | 0.000 | 1(0.919,1.087) | 0.991 |
| LDL-c (mmol/L) | 0.053 | 1.055(0.99,1.124) | 0.098 | 0.042 | 1.042(0.98,1.109) | 0.187 |
| TG (mmol/L) | -0.094 | 0.91(0.832,0.996) | 0.041 | -0.088 | 0.916(0.839,1) | 0.050 |
| ACR (mg/g) | 0.395 | 1.484(1.37,1.608) | ＜0.001 | 0.424 | 1.528(1.414,1.652) | ＜0.001 |
| eGFR (mL/min/1.73m^2^) | -0.067 | 0.935(0.851,1.026) | 0.158 | -0.034 | 0.966(0.883,1.058) | 0.457 |
